# Supplementary material for: Frequency of the Main Human Leukocyte Antigen A, B, DR, and DQ Loci Known to Be Associated with the Clearance or Persistence of Hepatitis C Virus Infection in a Healthy Population from the Southern Region of Morocco: A Preliminary Study
Source: Diseases. 2024 May 16;12(5):106. doi: 10.3390/diseases12050106 (PMC11120154; doi:10.3390/diseases12050106)
Supplement: Supplementary file 1 [file diseases-12-00106-s001.zip › diseases-2853506-supplementary.pdf]

Supplementary Materials

Table S1. Frequency of HLA-A loci by gender.

HLA-A Locus	**Female (n=329, 658 allele)**		**Male (n=355, 710 allele)**	
	**Allele group N**	**Allele Frequency (%)**	**Allele group N**	**Allele Frequency (%)**
**A1**	81	**12.31**	78	**11**
**A2**	155	**23.6**	164	**23.1**
**A3**	62	**9.42**	60	**8.45**
**A9**	0	-	3	0.42
**A10**	4	0.61	1	0.14
**A11**	24	3.65	26	3.66
**A19**	0	-	1	0.14
**A23**	46	7	63	8.87
**A24**	50	7.6	59	8.31
**A25**	1	0.15	1	0.14
**A26**	16	2.43	20	2.82
**A28**	7	1.06	4	0.56
**A29**	17	2.58	26	3.66
**A30**	35	5.32	54	7.61
**A31**	6	0.91	11	1.55
**A32**	28	4.26	22	3.1
**A33**	27	4.1	28	3.94
**A34**	15	2.28	16	2,25
**A36**	3	0.46	0	-
**A66**	7	1.06	6	0.85
**A68**	53	8.05	54	7.61
**A69**	3	0.46	0	-
**A74**	5	0.76	6	0.85
**A80**	13	1.98	7	1

Table S2. Frequency of HLA-B locus allele groups by gender.

**HLA-B Locus**	**Female (n=329, 658 allele)**		**Male (n=355, 710allele)**	
	Allele group N	Allele Frequency (%)	Allele group N	Allele Frequency (%)
**B2**	0	-	1	0.14
**B7**	30	4.56	**48**	**6.76**
**B8**	**48**	**7.29**	42	5.92
**B12**	1	0.15	0	-
**B13**	8	1.22	6	0.85
**B14**	30	4.56	29	4.08
**B15**	9	1.37	14	1.97
**B17**	4	0.61	7	1
**B18**	28	4.26	30	4.23
**B21**	2	0.3	2	0.28
**B27**	18	2.74	32	4.51
**B35**	42	6.38	36	5.07

**B37**	5	0.76	4	0.56
**B38**	15	2.28	28	3.94
**B39**	12	1.82	15	2.11
**B40**	20	3.04	14	1.97
**B41**	14	2.13	19	2.68
**B42**	12	1.82	17	2.39
**B44**	**53**	**8.05**	**73**	**10.28**
**B45**	38	5.78	44	6.2
**B47**	1	0.15	5	0.7
**B49**	**54**	**8.21**	34	4.79
**B50**	38	5.78	33	4.65
**B51**	**72**	**10.94**	**64**	**9,01**
**B52**	5	0.76	12	1.69
**B53**	17	2.58	19	2.68
**B55**	2	0.3	1	0.14
**B56**	1	0.15	2	0.28
**B57**	22	3.34	9	1.27
**B58**	21	3.19	36	5.07
**B62**	1	0.15	2	0.28
**B63**	18	2.74	11	155
**B64**	2	0.3	0	-
**B65**	0	-	1	0.14
**B70**	1	0.15	0	-
**B71**	0	-	1	0.14
**B72**	10	1.52	13	1.83
**B73**	1	0.15	0	-
**B78**	3	0.46	6	0.85

Table S3. Frequency of HLA-DRB1 allele groups by gender.

**HLA-DRB1 Locus**	**Female (n=166, 332allele)**		**Male (n=157, 314allele)**	
	Allele group N	Allele Frequency (%)	Allele group N	Allele Frequency (%)
**DRB1*01**	28	8.43	20	6.37
**DRB1*03**	67	**20.18**	57	**18.15**
**DRB1*04**	41	**12.35**	41	**13.06**
**DRB1*07**	56	**16.87**	40	**12.74**
**DRB1*08**	7	2.11	9	2.87
**DRB1*09**	4	1.2	6	1.91
**DRB1*10**	3	0.9	3	0.96
**DRB1*11**	36	10.84	29	9.24
**DRB1*12**	0	-	4	1.27
**DRB1*13**	52	**15.66**	50	**15.92**
**DRB1*14**	2	0.6	4	1.27
**DRB1*15**	36	**10.84**	50	**15.92**
**DRB1*16**	0	-	1	0.32

Table S4. Frequency of HLA-DQB1 allele groups by gender.

**HLA-DQB1 Locus**	**Female (n=166, 332 alleles)**		**Male (n=157, 314 alleles)**	
	Allele group N	Allele Frequency (%)	Allele group N	Allele Frequency (%)
**DQB1*02**	120	**36.14**	94	**29.94**
**DQB1*03**	80	**24.1**	75	**23.89**
**DQB1*04**	19	5.72	16	5.1
**DQB1*05**	44	13.25	40	12.74
**DQB1*06**	69	**20.78**	88	**28.03**
**DQB1*07**	0	-	1	0.32
